# Piperine Enhances the Antimalarial Activity of Curcumin in *Plasmodium berghei* ANKA-Infected Mice: A Novel Approach for Malaria Prophylaxis

**DOI:** 10.1155/2022/7897163

**Published:** 2022-09-05

**Authors:** Shafia Khairani, Nisa Fauziah, Hesti Lina Wiraswati, Ramdan Panigoro, Annas Salleh, Endang Yuni Setyowati, Afiat Berbudi

**Affiliations:** ^1^Doctoral Program in Medical Science, Faculty of Medicine, Padjadjaran University, Bandung, Indonesia; ^2^Veterinary Medicine Program, Faculty of Medicine, Padjadjaran University, Bandung, Indonesia; ^3^Department of Biomedical Sciences, Parasitology Division, Faculty of Medicine, Padjadjaran University, Bandung, Indonesia; ^4^Department of Biomedical Sciences, Biochemistry and Molecular Biology Division, Faculty of Medicine, Padjadjaran University, Bandung, Indonesia; ^5^Department of Veterinary Laboratory Diagnosis, Faculty of Veterinary Medicine, University Putra Malaysia, Serdang, Malaysia; ^6^Department of Animal Production, Faculty of Animal Husbandry, Padjadjaran University, Bandung, Indonesia

## Abstract

Malaria is a prevalent vector-borne infectious disease in tropical regions, particularly in the absence of effective vaccines and because of the emergence resistance of *Plasmodium* to available antimalarial drugs. An alternative strategy for malaria eradication could be the combination of existing compounds that possess antimalarial activity to target multiple stages of the parasite. This study evaluated the antimalarial activity of a combination of curcumin and piperine in mice. A total of 42 mice were assigned to six groups depending on the treatment administered: group I (normal group) with aquadest; group II (negative control) with 0.2 ml DMSO; group III received a standard malarial drug (artesunate 5 mg/kg BW); groups IV, V, and VI with curcumin 300 mg/kg BW, curcumin 300 mg/kg BW and piperine 20 mg/kg BW, and piperine 20 mg/kg BW, respectively. The antimalarial activity was evaluated using prophylactic assays in *Plasmodium berghei* ANKA-infected mice, including the percentage parasitemia, clinical signs, survival rate, serum biochemical analysis, parasitic load in the liver, and liver histopathology. All treatments showed significant (*p* < 0.05) antiplasmodial activity, with considerable parasite inhibition (>50%), curcumin 300 mg/kg BW (60.22%), curcumin 300 mg/kg BW, and piperine 20 mg/kg BW (77.94%) except for piperine 20 mg/kg BW (47.20%), eliciting greater inhibition relative to that of artesunate (51.18%). The delayed onset of clinical symptoms and prolonged survival rate were also significant (*p* < 0.05) in the combination of curcumin and piperine treated group. In addition, the low parasitic load in the liver and mild histopathological changes in the liver suggest that the combination of curcumin and piperine had synergistic or additive effects. These findings demonstrate the promising use of these combined compounds as a malarial prophylactic. Further studies were recommended to assess their clinical usefulness.

## 1. Introduction

Emerging resistance of the malarial parasite to frontline antimalarial drugs and the lack of effective drugs challenged the global malaria eradication strategy, consequently increasing the burden to human health. There were 241 million cases of malaria in 2020, with approximate 627,000 deaths worldwide [1]. Artemisinin-based combination therapies (ACTs) are to date the only beneficial treatment for malaria [2]. However, most of these therapies are stage-specific, exclusively targeting the blood stage rather than the liver stage [3].

*Plasmodium*, a unicellular haemosporidian, has an extremely complex life cycle in different hosts. Typically, an infected female *Anopheles* mosquito bites a human host and then inoculates approximately 100 sporozoites into the bloodstream or the lymphatic system, which migrate to the liver. The sporozoites invade and proliferate in hepatocytes [4], undergoing schizogony to generate merozoites. Subsequently, merozoites in the bloodstream rupture the host cells triggering the clinical signs of malaria [5]. Each phase of the life cycle has benefits and risks as a drug target, but targeting the hepatic phase may reduce malarial morbidity and mortality because it prefaces the actual symptomatic phase [6].

Currently, a combination of drugs with different modes of action or synergistic activity can overcome drug resistance [7]. The emergence of ACTs-resistant strains in Southeast Asia, Central Africa, Eastern India, and their inevitable dissemination to other areas have posed a challenge to the global malaria eradication programs [8–10]. Thus, the World Health Organization (WHO) recommends the identification of novel antimalarial drugs that possess acute multistage activity with low toxicity [11–13]. Hence, researchers have focused on exploring herbal prophylaxis used by rural societies to prevent malaria [14]. Herbs have been traditionally used as preventive medications as well as for health promotion [14, 15]. Indeed, over 1200 medicinal herbs have been used worldwide for the treatment of infectious diseases, including malaria [16].

Curcumin *(diferuloylmethane)* is a natural polyphenolic compound isolated from the rhizome of turmeric, *Curcuma longa* Linnaeus [17]. It is used as a flavoring, food color, or medicinal herb in traditional Indian medicine. Curcumin has numerous pharmacological, antioxidant, anti-inflammatory, and anticarcinogenic activities [18, 19]. Previous studies have reported the health beneficial effects of dietary polyphenols, e.g., curcumin from turmeric for preventive or therapeutic purposes in various types of cancer [19]. Furthermore, curcumin has well-known cytotoxic and parasiticidal effects on protozoan parasites in vitro (e.g., *Leishmania, Giardia, Trypanosoma,* and *Plasmodium falciparum*) [20–24]. Several studies have shown the beneficial impacts of curcumin as an antimalarial agent. For example, curcumin plays a role in disrupting *Plasmodium* organelles such as apicoplast, microtubules, and *PfATP6* as well as affecting parasite chromatin modification through *HAT* inhibition [25–27]. In addition, curcumin may promote the immune response against *Plasmodium* via increasing the reactive oxygen species, activating *PPARγ/Nrf2*, and upregulating CD36 expression in monocytes or macrophages to phagocytose parasite-infected erythrocytes [28]. Furthermore, curcumin inhibits glycogen synthase kinase-3*β* (GSK3*β*), which affects the production of the proinflammatory cytokines by inhibiting the transcriptional activity of NF-*κ*B [29]. In a murine model, curcumin also demonstrated potent activity against *Plasmodium berghei*, acting synergistically with artemisinin [27, 30]. Nonetheless, the poor bioavailability of curcumin due to expansive intestinal and hepatic metabolism along with rapid elimination restricts its clinical use [31]. However, the absorption, distribution, metabolism, excretion, and toxicity (ADMET) of a therapeutic [32] could be achieved by combining with bioenhancers like piperine.

Piperine is a natural alkaloid isolated from black pepper (*Piper nigrum*) [33]. It is widely used as a preservative and seasoning in diets, medical procedures (to cure intermittent fever, colds, asthma, diarrhea, colic pain, cholera, and malaria), in perfumery, and even as an insecticide [33, 34]. The ethyl acetate extract of *Piper nigrum* has promising antiplasmodial activity, with IC_50_ values of 12.5 ± 0.37 and 12.0 ± 0.6 g/mL in *Plasmodium falciparum* 3D7 and INDO strains, respectively [35]. In addition, *Piper nigrum*, an antimalarial, exhibits a wide range of therapeutic index with low toxicity (TC_50_ = 87.0 g/ml) [36]. Recently, oral administration of piperine 40 mg/kg BW in curative and prophylactic tests exhibited the parasitemia chemosuppression of 79.21% and 58.8% (*p* < 0.05), respectively, prolonged survival rate compared with the negative control group, and an ability to protect vital organs (i.e., lungs, liver, spleen, and kidneys) from damage [37]. Furthermore, the combination of piperine and curcumin can enhance the bioavailability of curcumin in human and animal models [38, 39].

Traditional healers understand that malaria prophylaxis should prevent the onset of clinical features (e.g., intermittent fever, headache, and cold) during the malaria transmission season [40]. The traditional prophylactic use of medicinal herbs that possess acute multistage activity is available from limited studies [40]. Recent studies reviewed that the curcumin and piperine combination possibly has prophylactic activity [39]; however, it must be proven empirically in animal models. Therefore, this study was designed to determine the prophylactic activity of the combined curcumin and piperine as antimalarial in *Plasmodium berghei* ANKA-infected mice.

## 2. Materials and Methods

### 2.1. Experimental Animals and Parasites

Male Swiss Webster mice weighing 25–30 g at 8–12 weeks of age were utilized to understand the prophylactic activity of curcumin and piperine combination. All animals were maintained at 20 ± 2°C with a 12 h light/dark cycle and provided standard feed and water ad libitum. The mice were acclimatized for one week prior to the experiment. The *Plasmodium berghei* ANKA strain was obtained from the Eijkman Institute of Biology Molecular, Jakarta, Indonesia, and continuously maintained by a serial passage on a weekly basis. The donor mice were sacrificed, and blood was collected by cardiac puncture before 0.2 ml of blood suspension containing 1 × 10^6^*P. berghei* ANKA-infected RBC was inoculated intraperitoneally.

### 2.2. Prophylactic Study

The prophylactic activity test of the combination of curcumin and piperine was adapted from Peter's method as established in our previous work [37]. Curcumin (catalog no. C7727, ≥80%), piperine (catalog no. P49007, ≥97%), and artesunate (catalog no. A3731) were obtained from Sigma-Aldrich Inc (United States). All compounds were dissolved in DMSO (dimethyl sulfoxide) and administered by oral gavage. A total of 42 mice were assigned to six groups (*n* = 7 per group): group I (normal) was administered aquadest (distilled water) which represented the normal condition of the experimental animals; group II (negative control) was administered 0.2 ml DMSO; group III was administered a standard malarial drug (artesunate 5 mg/kg BW); and groups IV, V, and VI were administered curcumin 300 mg/kg BW, curcumin 300 mg/kg BW and piperine 20 mg/kg BW, and piperine 20 mg/kg BW, respectively. All treatments were provided daily for four consecutive days, and all mice were inoculated with parasites on day 5. Forty-two hours after *Plasmodium berghei* ANKA inoculation, two mice in each group were sacrificed with ketamine-xylazine. Blood samples were collected using a sterile tube for serum separation by centrifugation at 3,000 rpm for 20 min. The left lateral lobe of the liver was excised and perfused with phosphate-buffered saline (PBS) for quantitative real-time polymerase chain (qRT-PCR), while the other part of the liver was preserved in 10% formalin for histopathology and immunohistochemistry.

### 2.3. Determination of Parasitemia

Blood was obtained by trimming the tip of the tail on day 8 and smeared on a microscope slide (thin blood smear) and then fixed with absolute methanol for 10 s. After fixation, the slides were dried and stained with 10% Giemsa stain for 15 min, rinsed with running water, and dried at room temperature. The parasite-infected red blood cells were quantified using a microscope with oil immersion at 100× magnification. The percentage of parasitemia was determined using the formula described by Kalra et al. [41]:

(1) $$\begin{array}{c} \%\text{ parasitemia}=\frac{\text{number of parasitized RBC}}{\text{total number of RBC}}\times 100\%. \end{array}$$

The percentage of inhibition of the parasite was calculated via the following formula:

(2) $$\begin{array}{c} \text{\% inhibition}=\frac{\text{mean \% parasitemia of untreated group}-\text{mean \% parasitemia of treated group}}{\text{mean \% parasitemia of untreated group}}\times 100\%\text{.} \end{array}$$

### 2.4. Determination of Clinical Signs and Survival Rate

The *P. berghei* ANKA-infected mice were observed routinely (2 times daily) and scored for typical symptoms (ruffling hair, hunching, wobbly gait, limb paralysis, convulsions, coma, and eventually death) [42]. Each sign was given a score of 1. Animals with severe clinical symptoms (accumulative score of ≥4) were sacrificed with cervical dislocation according to the guideline for the euthanasia of animals [43]. For each tagged experimental mouse, clinical symptoms and survival rates were recorded daily.

### 2.5. Serum Biochemical Analysis

Alanine aminotransferase (ALT) and aspartate aminotransferase (AST) were quantified in mouse serum using a commercially available kit (Cat. no. AL1205 and AS1204, Randox Laboratories, UK) according to the manufacturer's instructions.

### 2.6. RNA Extraction and Gene Expression Analysis

The RNA was extracted from the liver using the Quick-RNA™ Miniprep Kit (R1054, Zymo Research, USA) according to the manufacturer's instructions. The expression of the *18S rRNA* gene, a marker of *Plasmodium berghei* ANKA, was analyzed using qRT-PCR. At least 13 *µ*l of total RNA was reverse transcribed using the SensiFAST™ cDNA Synthesis Kit (BIO-65053, Bioline Ltd., UK) according to the manufacturer's instructions. PCR was performed using the SensiFAST™ SYBR® No-ROX Kit (BIO-98005, Bioline Ltd) according to the manufacturer's instructions. Quantitative real-time PCR was performed at 95°C for 2 min, followed by at least 40 cycles at 95°C for 5 s and 60°C for 15 s. The primer sequences were Pb *18S rRNA* forward: AAG CAT TAA ATA AAG CGA ATA CAT CCT TAC, Pb *18S rRNA* reverse: GGA GAT TGG TTT TGA CGT TTA TGTG and mouse *β-actin* forward: GGC TGT ATT CCC CTC CAT CG, mouse *β-actin* reverse: CCA GTT GGT AAC AAT GCC ATGT [44]. Gene expression was analyzed using the 2^−ΔΔCt^ method, normalized to the housekeeping gene mouse *β-actin* gene, and presented as a fold change relative to the control group.

### 2.7. Histopathological Examination

The paraffined organ was sliced to 3-4 *µ*m thickness and then H&E stained. Histopathological slides were observed using a light microscope (Olympus BX 53 with camera Olympus DP 23, Japan). Tissue micrographs were generated using a 10× and 40× objective lens for further analysis. Histopathological changes were recorded using a standard nonlinear semiquantitative scoring system and a scale from 0 to 5 adapted from Shackelford et al. [45]. Significant findings were scored 0 (normal architecture), 1 for mild changes that could be observed by light microscopy (<10% of affected tissue), 2 for mild changes easily detectable but not a primary feature (<20%), 3 for moderate changes expected to associate with altered organ function, 4 for severe changes in up to 75% of the tissue, and last, 5 if the entire tissue was affected leading to altered organ functionality. Changes in the control and treatment groups were constantly compared and recorded.

### 2.8. Immunohistochemistry

Localization of CD68 was performed by immunostaining of the liver sections using the streptavidin-biotinylated horseradish peroxidase method (Thermo Fisher Scientific, United States). Endogenous peroxidase activity was inhibited by hydrogen peroxide for 5–10 min; then, the sections were washed twice in PBS for 5 min. The ultra V block was applied for 5 min to block nonspecific background staining. Sections were rinsed twice for 5 min and then incubated with primary antibody (anti-CD68 ab125212, Abcam-US; diluted 1 : 400) according to the manufacturer's instructions. The immunohistochemistry slides were observed blind using a trinocular clinical light microscope (Olympus BX 53 with camera Olympus DP 23, Japan). For the CD68 staining, six microscopical views (40× magnification) of each liver were obtained. Image J software was used to analyze CD68-positive (brown) pixels as well as total unstained tissue pixels of each microscopical picture. Subsequently, these data were used to calculate the percentage of the CD68-positive area.

### 2.9. Ethical Approval

This study was approved by the research ethics committee, Faculty of Medicine, Padjadjaran University, Bandung, Indonesia (no.: 1045/UN.6.KEP/EC/2020) and conducted according to the Animal Use Guidelines.

### 2.10. Statistical Analysis

Data are expressed as mean ± standard error of the mean (SEM). The mean differences of the measured parameters were compared by two-way analysis of variance (ANOVA) using GraphPad Prism Windows version 9, followed by post-hoc (Tukey method) multiple comparisons. A *p* < 0.05 was considered statistically significant.

## 3. Results

### 3.1. A Combination of Curcumin and Piperine Impedes Parasitemia

**Figure 1:**
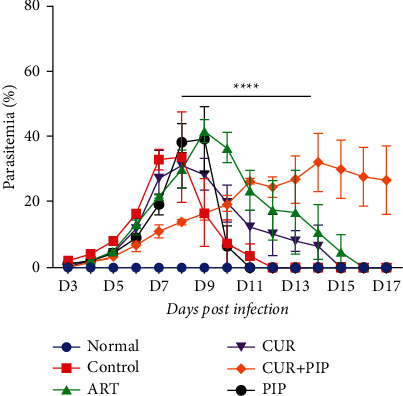
Percentage of parasitemia after curcumin and piperine administration in *Plasmodium berghei* ANKA-infected mice (*n* = 5 per group). Data are presented as mean ± SEM. ^*∗∗∗∗*^*P* < 0.0001. ART, artesunate 5 mg/kg BW; CUR, curcumin 300 mg/kg BW; CUR + PIP, curcumin 300 mg/kg BW and piperine 20 mg/kg BW; PIP, piperine 20 mg/kg BW.

The prophylactic activity of the combination of curcumin and piperine on parasitemia is presented in Figure 1, showing significant inhibition of parasitemia (*p* < 0.0001) compared to that of the negative controls. The peak of parasitemia in negative controls and the curcumin-treated group occurred on day 8, while the artesunate and piperine groups reached peak parasitemia on day 9. Interestingly, the percentage of parasitemia in the combination of curcumin and piperine-treated group increased more slowly than that of the other groups peaking on day 14.

### 3.2. A Combination of Curcumin and Piperine Improves Clinical Sign and Prolongs Survival Rate

**Figure 2:**
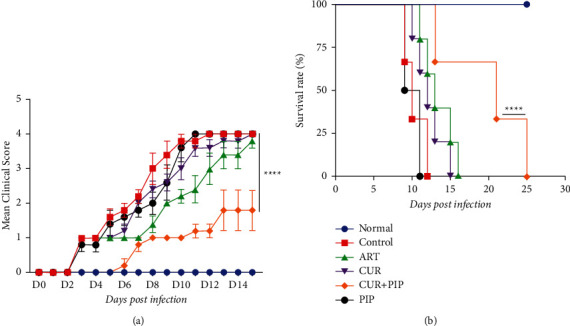
(a) Clinical sign scores and (b) survival rate after curcumin and piperine administration in *P. berghei* ANKA-infected mice (*n* = 5 per group). Data are presented as mean ± SEM. ^*∗∗∗∗*^*P* < 0.0001. ART, artesunate 5 mg/kg BW; CUR, curcumin 300 mg/kg BW; CUR + PIP, curcumin 300 mg/kg BW and piperine 20 mg/kg BW; PIP, piperine 20 mg/kg BW.

The mean clinical sign score in the combination of curcumin and piperine group was significantly different (*p* < 0.0001) compared to the negative control, artesunate-treated group, curcumin (300 mg/kg BW), and piperine (20 mg/kg BW) (Figure 2(a)), with the combination of curcumin and piperine delaying the onset of clinical signs (D6-D12). It is in line with the survival rate of mice. The administration of combined curcumin and piperine showed a prolonged survival rate (*p* < 0.0001) compared to that of the negative control, artesunate-treated, curcumin (300 mg/kg BW), and piperine (20 mg/kg BW) groups (Figure 2(b)). All mice in the negative control group died on day 12.

### 3.3. A Combination of Curcumin and Piperine Prevents Serum Biochemical Escalation and Histopathological Changes in the Liver

**Figure 3:**
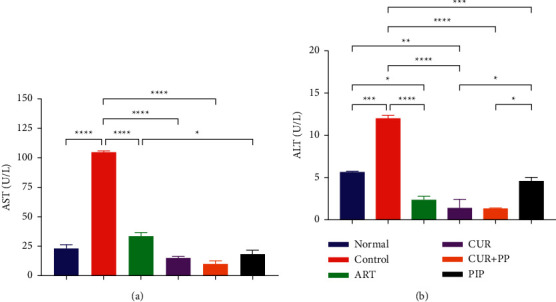
(a) Alanine aminotransferase (AST) and (b) aspartate aminotransferase (ALT) after curcumin and piperine administration in *P. berghei* ANKA-infected mice (*n* = 2 per group). The data are presented as mean ± SEM. ^*∗∗∗∗*^*P* < 0.0001; ^*∗∗∗*^*p* < 0.001; and ^*∗*^*p* < 0.01. ART, artesunate 5 mg/kg BW; CUR, curcumin 300 mg/kg BW; CUR + PIP, curcumin 300 mg/kg BW and piperine 20 mg/kg BW; PIP, piperine 20 mg/kg BW.

**Figure 4:**
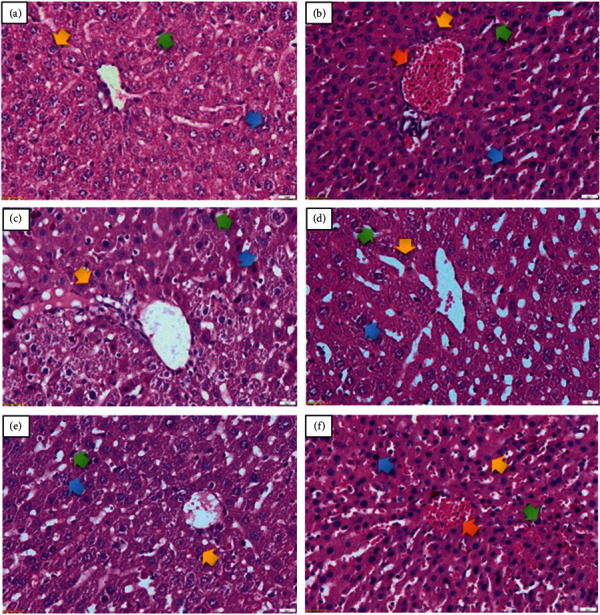
Normal hepatic architecture—normal hepatocytes and sinusoids (a); the liver from the untreated group showing severe congestion, necrosis, Kupffer cell hyperplasia, and moderate inflammation (b); ART–mild inflammation and necrosis (c); CUR–necrosis, mild inflammation, sinusoidal dilatation, and Kupffer cell hyperplasia (d); CUR + PIP–the moderate renewal of the degenerated hepatocytes, mild inflammation, and sinusoidal dilatation (e); PIP–moderate necrosis, Kupffer cell hyperplasia, mild sinusoidal congestion, and inflammation (f); Yellow arrow, normal cells; blue arrow, necrotic cells; green arrow, Kupffer cells; orange arrow, congestion. Magnification: 40×. ART, artesunate 5 mg/kg BW; CUR, curcumin 300 mg/kg BW; CUR + PIP, curcumin 300 mg/kg BW and piperine 20 mg/kg BW; PIP, piperine 20 mg/kg BW.

The effect of combined curcumin and piperine on the liver function was observed by determining the AST and ALT levels of the treated mice groups compared with the negative control group. All the treated groups exhibited lower AST and ALT levels than the negative controls, with the combination of curcumin and piperine showing the best overall performance (Figures 3(a) and 3(b)). This is in line with the liver histopathological changes (Figures 4(a)–4(f)). The malaria parasite causes histopathological damage to the liver, including hepatic necrosis, Kupffer cell hyperplasia, portal inflammation (hepatitis), and an excessive or abnormal accumulation of blood in the blood vessels (congestion). The administration of the curcumin and piperine combination reduced the pathologies caused by the malarial parasites more visibly compared to that of the control group (Table 1) but was not differently significant from artesunate.

### 3.4. A Combination of Curcumin and Piperine Suppresses the Parasitic Load and CD68 Phagocytic Cell Aggregation in the Liver

**Figure 5:**
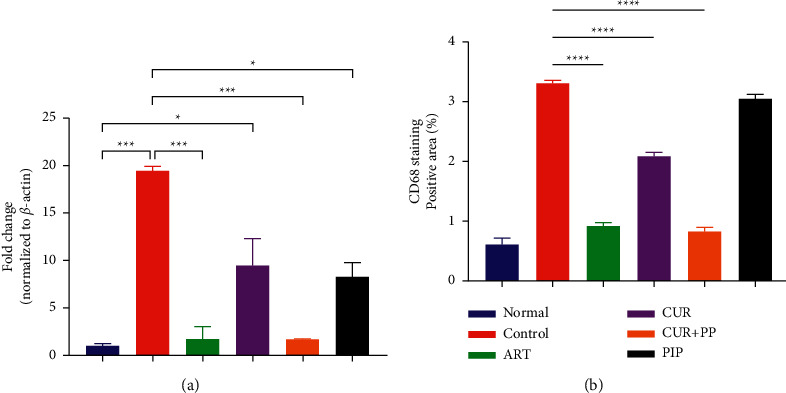
(a) Relative parasitic load and (b) quantification of the percentage CD68-positive area after curcumin and piperine administration in *P. berghei* ANKA-infected mice (*n* = 2 per group). The data are presented as mean ± SEM. ^*∗∗∗∗*^*P* < 0.0001; ^*∗∗∗*^*p* < 0.001; and ^*∗*^*p* < 0.01. ART, artesunate 5 mg/kg BW; CUR, curcumin 300 mg/kg BW; CUR + PIP, curcumin 300 mg/kg BW and piperine 20 mg/kg BW; PIP, piperine 20 mg/kg BW.

**Figure 6:**
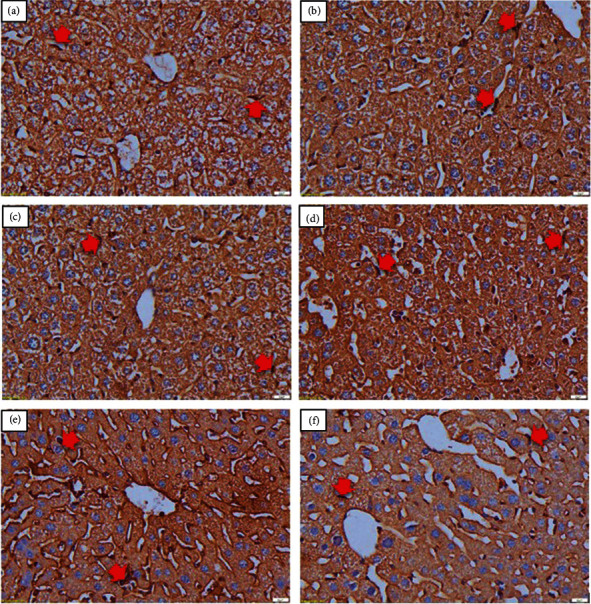
Immunohistochemistry examination. Normal distribution of Kupffer cells in normal liver sinusoid (a); the liver from the untreated group showing aggregated and scattered Kupffer cells, especially in the central venous area (++++) (b); ART: liver from mice receiving artesunate showing normal distribution of Kupffer cells (c); CUR: moderate decrease in the foci of aggregated Kupffer cells but still more than the normal group (++) (d); CUR + PIP: a mild increase in scattered Kupffer cells but without aggregations (++) (e); and PIP: a moderate increase in Kupffer cells aggregation (+++) (f); Red arrow, Kupffer cells. Magnification: 40×. ART, artesunate 5 mg/kg BW; CUR, curcumin 300 mg/kg BW; CUR + PIP, curcumin 300 mg/kg BW and piperine 20 mg/kg BW; PIP, piperine 20 mg/kg BW.

All treated groups significantly reduced the parasitic load in the liver compared to the negative control. Interestingly, the combination of curcumin and piperine demonstrated comparable performance to artesunate (Figure 5(a)). It is in line with the immunohistochemical staining (CD68) of the liver. The normal group with anti-CD68 antibody shows the normal distribution of Kupffer cells in sinusoids. Interestingly, artesunate demonstrated a similar effect, whereas liver sections from untreated mice had scattered aggregations of Kupffer cells mainly around the central vein. The administration of curcumin alone showed a greater decrease in the foci of aggregated Kupffer cells compared to the normal group. The liver section from mice receiving the curcumin and piperine combination showed a slight increase in scattered Kupffer cells but a lack of aggregations (Figure 6(a)–6(f)). These data were confirmed by quantification of the CD68-positive area of these livers (Figure 5(b)).

## 4. Discussion

Malaria, a vector-borne disease, is still one of the most concerning infectious diseases in tropical regions worldwide. Although the causative parasites have been identified and effective drugs developed, this devastating disease is yet to be eliminated [46]. There are ongoing efforts to develop novel antimalarial drugs and effective vaccines; but to date, there is no single effective drug for the treatment of multidrug-resistant malaria [47]. Hence, the novel combination therapy that can inhibit multiple stages of parasite life cycle as well as having a broader mode of action is required. The potent and sustained antimalarial activity of curcumin [26, 27, 39, 48–50] and the antimalarial activity of piperine [37, 39, 51, 52] prompted us to assess the potential prophylactic activity of combined curcumin and piperine as an alternative strategy in the fight against malaria.

The prophylactic antiplasmodial model is one of the standard methods generally used for screening candidates that determine the percentage inhibition of parasitemia as the primary parameter. A percentage inhibition of parasitemia ≥10% relative to the negative control generally indicates that the test candidate is active [53]; hence, all the compounds evaluated in this study could be perceived as active antimalarial candidates. In addition, the antimalarial activity (using an in vivo model) is determined as moderate, good, or very good if a compound demonstrates percentage parasitemia inhibition ≥50% at a dose of 500, 250, and 100 mg/kg/day, respectively [54]. Based on this classification, artesunate, the standard drug for malaria, has proven to have very good antiplasmodial activity. Meanwhile, both curcumin (60.22%) and the combination of curcumin and piperine (77.94%) showed good antiplasmodial activity. Although the efficacy of malarial therapy is not only determined by the percentage of parasite inhibition, the profile of daily parasitemia should be considered. Interestingly, the drug combination demonstrated more inhibition than that using the single drugs alone, indicating that the combination of curcumin and piperine has a protective effect against *Plasmodium* infection.

In parallel with the lower levels of parasitemia in mice treated with combined curcumin and piperine, the clinical signs exhibited by this group were also lower than those of the negative control. The onset of clinical signs in malarial infection is an indication that parasites have infected the red blood cells [55], with severe clinical signs reflecting high levels of parasitemia [42]. Neurological symptoms commonly occur within 5–10 days of infection and can lead to death [56]. However, administration of combined curcumin and piperine demonstrated slow development of clinical signs compared to the negative control and prolonged the survival of the mice.

These results were confirmed by qRT-PCR in assessing the parasite load in the liver and immunohistochemistry for the localization of the CD68 phagocytic cells. All treatments prevented the escalation of the parasitic load in the liver compared to the negative control, with the combination of curcumin and piperine being the most effective treatment. Phagocytic cell (Kupffer cell) activation in the liver is commonly associated with the phagocytic response to infectious agents. Interestingly, administration of combined curcumin and piperine showed a moderate number of Kupffer cells compared to the negative control. The decrease in CD68 phagocytic cells after curcumin administration has been reported previously in the liver injury induced by acetaminophen [57]. Hence, this phenomenon might have occurred due to the hepatoprotective properties of curcumin or owing to the low amount of *Plasmodium* in the liver, thereby reducing the immune response in these tissues.

Hepatomegaly is a hallmark of malarial infection [58], triggering an increase in the AST and ALT levels as well as markers of the liver damage due to infection [59]. High AST and ALT levels were observed in the negative control and associated with severe histopathological changes in the liver [59]. However, the administration of the curcumin and piperine combination prevented the increase in AST and ALT as well as reduced the changes in liver histopathology. Only mild inflammation and slight necrosis were observed in the curcumin and piperine combination that may be attributed to the anti-inflammatory properties of curcumin and piperine [60, 61].

Generally, the prophylactic activity of these compounds may be due to inhibited proliferation of plasmodial parasites as a result of direct cytotoxicity [62]. Curcumin is a flavonoid and its metabolites have immunomodulatory effects, including activation of TLR2, increased IL-10, production of parasite antibodies, and stimulation of macrophages (M2) in murine models [63–65]. Curcumin has also been demonstrated to inhibit histone acetylation and generate reactive oxygen species that can be cytotoxic to *Plasmodium* [26]. While, piperine, an alkaloid, is well known for its potent antioxidant and hepatoprotective and good anti-inflammatory effects [66]. Previous studies suggested that piperine has curative and prophylactic activities against *Plasmodium* infection [37], may be due to its potential parasitic-killing ability demonstrated by other alkaloids such as quinine and chloroquine (acts as a blood schizonticide) [67]. In another in vivo study, a combination of piperine and curcumin was conducted by a curative assay, which reduced parasitemia in *P. chabaudi*-infected mice [48, 49]. The specific mechanism of action of curcumin and piperine as a malarial prophylactic remains still unclear. These compounds have different structures as well as modes of action; however, combining curcumin and piperine can improve the bioavailability of curcumin and shows synergistic effects [39], thus exhibiting good antiplasmodial and hepatoprotective activities. Although our findings indicate that the combination of curcumin and piperine possesses antimalarial properties and could be useful as prophylactic for malaria, further studies are needed to evaluate the specific mechanisms of action and pharmacokinetics. Nonetheless, this study confirms the potential use of herbal medicines as an effective approach for malaria control.

## 5. Conclusion

The combination of curcumin and piperine provided a good antimalarial activity with a synergistic effect on *Plasmodium berghei* ANKA-infected mice, including inhibition of parasitemia, delayed onset of clinical signs, and prolonged survival rate. In addition, the low parasitic load in the liver, lack of elevation in ALT and AST serum, and good histopathological features of the liver suggest that piperine may serve as a potential partner that can be combined with curcumin as malaria prophylaxis.

## Figures and Tables

**Table 1:** Semiquantitative analysis of liver histopathology.

Group	Histopathological parameters				Overall lesion scores
	Hyperplasia Kupffer cells	Sinusoid congestion	Portal inflammation	Necrosis	
Control	3.50 ± 0.50	3.50 ± 0.50	3.50 ± 0.50	4.00 ± 0.00	14.5/20
ART	1.00 ± 0.00	1.00 ± 0.00	1.00 ± 0.00	1.00 ± 0.00	4/20
CUR	2.50 ± 0.50	1.00 ± 0.00	1.50 ± 0.50	2.50 ± 0.50	7.5/20
CUR + PIP	1.50 ± 0.50	1.00 ± 0.00	1.00 ± 0.00	1.00 ± 0.00	4.5/20
PIP	3.50 ± 0.50	2.00 ± 0.00	2.00 ± 0.00	3.00 ± 0.00	10.5/20

The data are presented as mean ± SEM.

## Data Availability

The datasets used to support the findings of this study are included within the article.
